# Superior vena cava syndrome caused by a swollen absorbable haemostat after repair of ischaemic mitral regurgitation

**DOI:** 10.1186/1749-8090-9-1

**Published:** 2014-01-03

**Authors:** Koki Eto, Mitsuaki Matsumoto, Yoji Kubo, Reiko Kemmochi

**Affiliations:** 1 Department of Cardiovascular Surgery, Cardiovascular Center, Tsuyama Chuo Hospital, 1756, Kawasaki, Tsuyama City, Okayama 708-0841, Japan

**Keywords:** Surgicel, SVC syndrome, Haematoma, External compression, Cardiovascular surgery

## Abstract

Surgicel, an absorbable haemostat, is widely used in cardiovascular surgery. An 81-year-old woman, who was diagnosed with ischaemic mitral regurgitation, underwent mitral valve plasty and coronary artery bypass grafting. On postoperative day two, her superior vena cava (SVC) pressure gradually rose to 38 mmHg and she developed low output syndrome. Emergent surgery revealed that the cause of SVC syndrome was external compression from a haematoma at the posterior surface of the SVC, which formed around the Surgicel.

## Background

Haemostatic agents are quite useful in almost all surgeries. An absorbable haemostat is also effective in controlling oozing of suture lines or stripped surfaces. Surgicel (Ethicon, North Ryde, NSW, Australia), an absorbable sheet of oxidized cellulose polyanhydroglucuronic acid polymer, has been used in numerous cardiovascular surgeries; however, recent reports have revealed that Surgicel can disturb organ function in several contexts [1-4]. We report a case of superior vena cava (SVC) syndrome due to compression by an haematoma formed around swollen Surgicel.

## Case presentation

**Figure 1:**
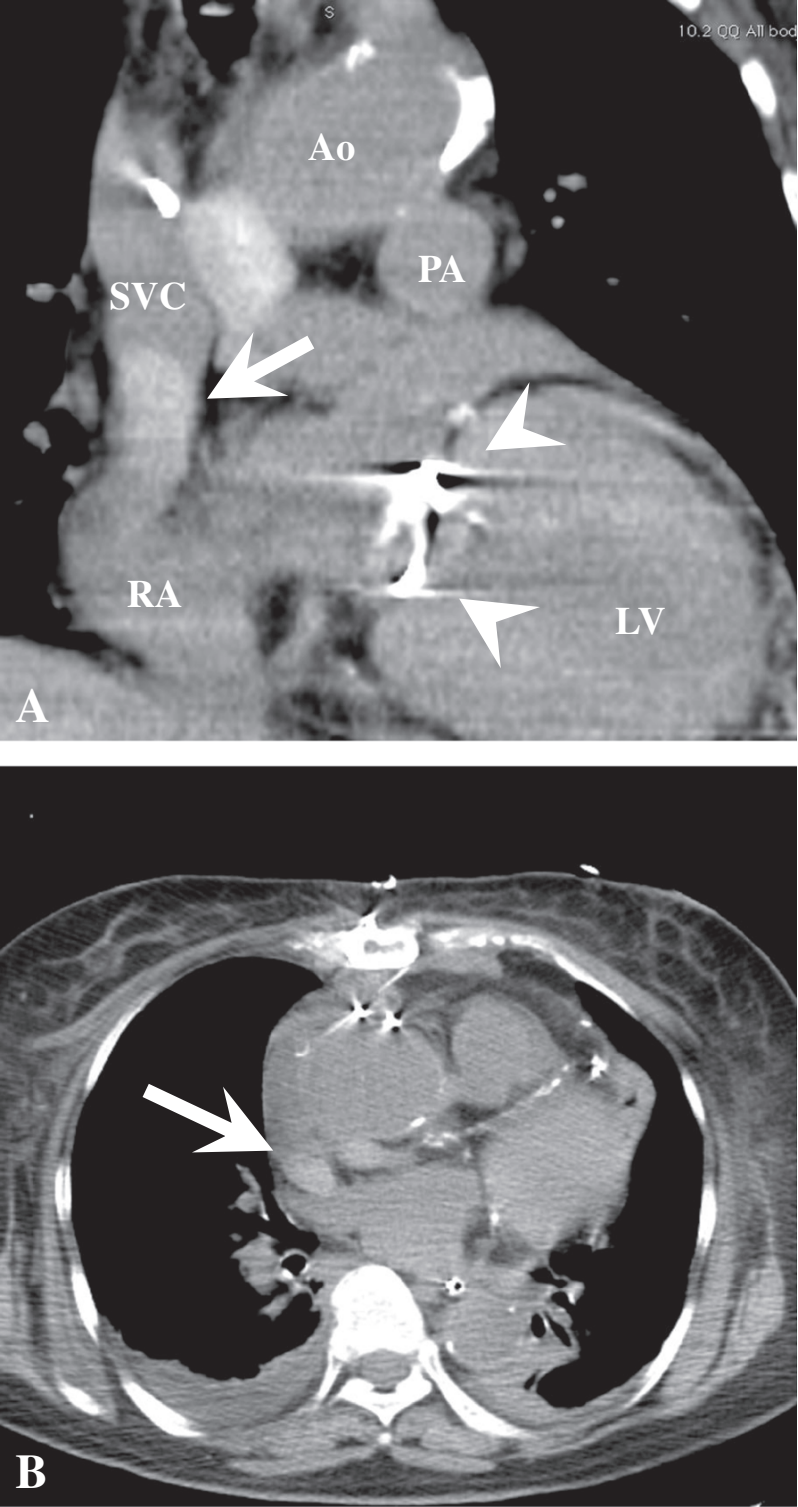
**Planar computed tomography. A**, Coronal view. **B**, Axial view. A high density area (arrow) confined at the terminal groove was observed. A mitral ring was observed (arrowhead). Ao: aorta, PA: pulmonary artery, LV: left ventricle, RA: right atrium, SVC: superior vena cava.

**Figure 2:**
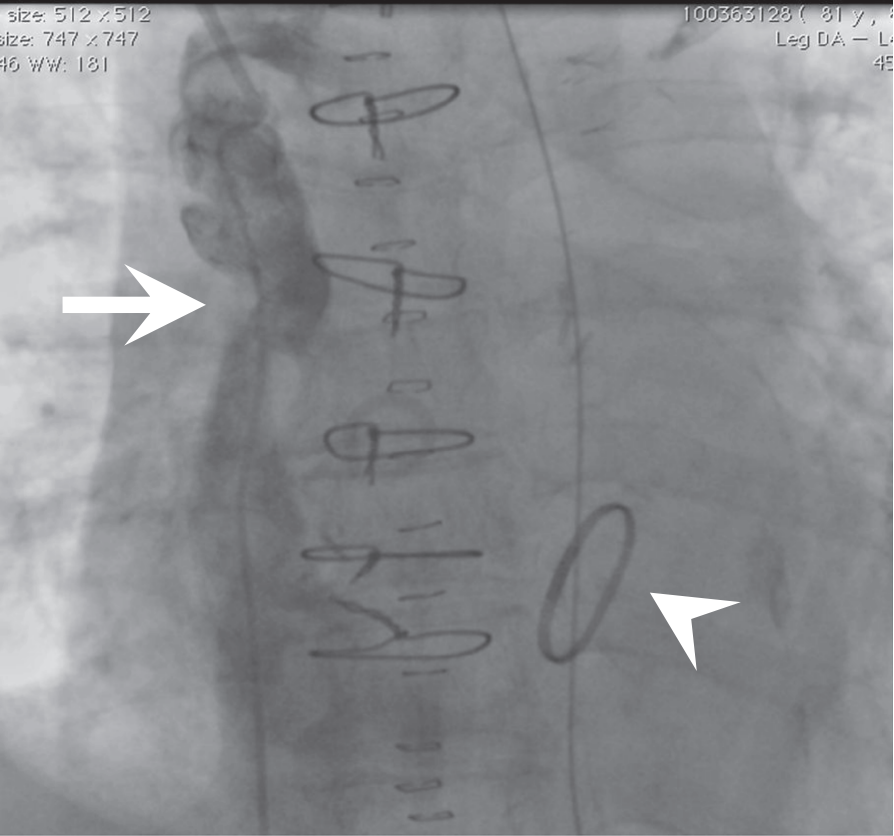
**Superior vena cava (SVC) angiography.** Stenosis at the SVC (arrow) and a mitral ring (arrowhead) were observed.

An 81-year-old Japanese woman had long been followed for chronic heart failure. She complained of dyspnoea during light exercise that was gradually worsening. Subsequent coronary angiography demonstrated three-vessel disease. Owing to chronic ischaemia, echocardiography revealed diffuse hypokinesis of the left ventricle and severe mitral regurgitation associated with the tethering of mitral leaflets due to left ventriclar dilatation. The patient underwent mitral valve plasty (MVP) and coronary artery bypass grafting (CABG) simultaneously under a standard medial sternotomy and a cardiopulmonary bypass (CPB) established by cannulation of the ascending aorta, SVC, and inferior vena cava. MVP was performed using a 24-mm Carpenter-Edwards Physio Ring (Edwards Lifesciences, Irvine, CA, USA) and a papillary muscle (PM) approximation, which was performed by suturing the tops of both anterolateral and posteromedial PMs using CV-4 with felt strips from the left atrium. CABG to the left ascending artery, left circumflex artery, and right coronary artery was performed using the harvested left internal thoracic artery and saphenous vein grafts. On postoperative day two, she suddenly showed a moon face appearance with an increased SVC pressure of 38 mmHg. Although echocardiography did not show evidence of tricuspid regurgitation or SVC occlusion, computed tomography (CT) revealed the possibility of a thrombus in the SVC (Figure 1), and SVC angiography showed severe stenosis at the terminal groove (Figure 2). Since she showed low output syndrome and the thrombus might have caused serious pulmonary thrombosis, emergent redo surgery was performed. The anterior surface of the SVC appeared intact; however, a haematoma was observed in the posterior side of the SVC, which caused severe stenosis of the SVC trunk. Careful observation revealed that the core of the haematoma consisted two pieces of Surgicel, which had been left during the first operation to control the oozing of the surface of the anterior side of the right pulmonary artery. The postoperative bleeding had continued and gathered around the haemostats. After removing the haematoma, the SVC pressure decreased immediately to 11 mmHg. The patient’s postoperative course was uneventful.

## Discussion

Surgicel, an absorbable sheet of oxidized cellulose polyanhydroglucuronic acid polymer, has been used in many surgical fields. Surgicel is used at surgical oozing sites, physically promoting coagulation and producing an artificial clot [1,2].

Although Surgicel is often left in surgical sites for effective postoperative haemostasis, previous reports have revealed that a swollen Surgicel can compress and obstruct the function of neighbouring organs. Dogan et al. reported a case of paraplegia following left-sided thoracotomy, suggesting that Surgicel may migrate into the vertebral canal and produce haematomas that result in spinal cord compression [1]. Another report has described the complications associated with Surgicel-produced haematomas, including hemiparesis after trigeminal decompression [2].

As in other surgical specialties, several cases of Surgicel-related complications in cardiovascular surgery have been reported [3-5]. A recent report showed that Surgicel caused severe stenosis of a pulmonary homograft when Surgicel was placed around the suture lines of the pulmonary artery [4]. However, no cases of postoperative SVC syndrome caused by Surgicel have been reported.

In most cases, SVC syndrome is associated with lung cancer, other tumours, SVC catheters, and venous thrombosis, whereas postoperative SVC syndrome in cardiovascular surgery is rare. In our case, the patient developed SVC syndrome suddenly on postoperative day two. Although her international normalized ratio 1.4 and activated partial thromboplastin time 38.2 seconds were within nornal limits, her clinical course and images on CT and angiography suggested the possibility of catheter-associated SVC thrombosis or stenosis caused by CPB cannulation; however, the emergent redo surgery revealed that the haematoma in the posterior side of the SVC was compressing the SVC externally and swollen Surgicel had enhanced compression by the haematoma. This was a finding that is clearly different from compression secondary to Surgicel granuloma formation occurring at a later stage of surgery. Furthermore, in spite of no evidence of the injury of the right pulmonary artery, a very small amount of bleeding had continued from the sternum. In the elderly, the density of the sternum is significantly low, which may promote bleeding from the bone marrow postoperatively, thus resulting in development of “Surgiceloma” [2].

Surgicel is quite useful in many haemostatic situations, whereas the Surgicel left in surgical sites can produce a postoperative “Surgiceloma” that can be an external compressor, particularly in lower pressure systems or organs such as atriums and veins. Our experience may impress upon surgeons the importance of appropriate use of haemostatic agents.

## Conclusions

Surgeons should consider the possibility of postoperative compression by haemostatic agents swollen with blood, specially in lower pressure systems or organs.

## Consent

Written informed consent was obtained from the patient for publication of this case report and accompanying images. A copy of the written consent is available for review by the Editor-in-Chief of this journal.

## Abbreviations

SVC: Superior vena cava; MVP: Mitral valve plasty; CABG: Coronary artery bypass grafting; CPB: Cardiopulmonary bypass; PM: Papillary muscle; CT: Computed tomography.

## Competing interests

The authors declare that they have no competing interests.

## Authors’ contributions

KE wrote the manuscript and collected references. MM was the chief surgeon and commented to this manuscript. YK and RK were primarily involved in the clinical and scientific discussion of the case. All authors read and approved the final manuscript.
